# Validity and reliability of telephone administration of the patient-specific functional scale for the assessment of recovery from snakebite envenomation

**DOI:** 10.1371/journal.pntd.0007935

**Published:** 2019-12-13

**Authors:** Rebecca G. Theophanous, Joao Ricardo Nickenig Vissoci, Fan Hui Wen, S. Michelle Griffin, Victoria E. Anderson, Michael E. Mullins, Nicklaus P. Brandehoff, Eugenia B. Quackenbush, Sean P. Bush, Eric A. Toschlog, Spencer C. Greene, Kapil Sharma, Kurt Kleinschmidt, Nathan P. Charlton, S. Rutherfoord Rose, Richard Schwartz, Brandon Lewis, Eric J. Lavonas, Charles J. Gerardo

**Affiliations:** 1 Division of Emergency Medicine, Department of Surgery, Duke University School of Medicine, Durham, NC, United States of America; 2 Duke Global Health Institute, Duke University, Durham, NC, United States of America; 3 Hospital Vital, Instituto Butantan, São Paulo, Brazil; 4 Rocky Mountain Poison and Drug Center, Denver Health and Hospital Authority, Denver, CO, United States of America; 5 Division of Emergency Medicine, Washington University School of Medicine, St. Louis, MO, United States of America; 6 Department of Emergency Medicine, University of California San Francisco Fresno, Fresno, CA, United States of America; 7 Department of Emergency Medicine, University of North Carolina School of Medicine, Chapel Hill, NC, United States of America; 8 Department of Emergency Medicine, Brody School of Medicine at East Carolina University, Greenville, NC, United States of America; 9 Department of Surgery, Brody School of Medicine at East Carolina University, Greenville, NC, United States of America; 10 Henry J. N. Taub Department of Emergency Medicine, Baylor College of Medicine, Houston, TX, United States of America; 11 Department of Emergency Medicine, University of Texas Southwestern Medical Center, Dallas, TX, United States of America; 12 Division of Medical Toxicology, University of Virginia, Charlottesville, VA, United States of America; 13 Department of Emergency Medicine, Virginia Commonwealth University, Richmond, VA, United States of America; 14 Department of Emergency Medicine and Hospital Services, Medical College of Georgia, Augusta, GA, United States of America; 15 Texas A&M Health Science Center, College Station, TX, United States of America; 16 Department of Emergency Medicine, Denver Health and Hospital Authority, Denver, CO, United States of America; 17 Department of Emergency Medicine, University of Colorado School of Medicine, Aurora, CO, United States of America; Faculty of Medicine, University of Kelaniya, SRI LANKA

## Abstract

**Objectives:**

Although more than 1.8 million people survive snakebite envenomation each year, their recovery is understudied. Obtaining long-term follow-up is challenging in both high- and low-resource settings. The Patient-Specific Functional Scale (PSFS) is an easily administered, well-accepted patient-reported outcome that is validated for assessing limb recovery from snakebite envenomation. We studied whether the PSFS is valid and reliable when administered by telephone.

**Methods:**

This is a secondary analysis of data from a randomized clinical trial. We analyzed the results of PSFS collected in-person on days 3, 7, 14, 21, and 28 and by telephone on days 10, 17, and 24. We assessed the following scale psychometric properties: (a) content validity (ceiling and floor effects), (b) internal structure and consistency (Cronbach’s alpha), and (c) temporal and external validity using Intraclass Correlation Coefficient (ICC). Temporal stability was assessed using Spearman’s correlation coefficient and agreement between adjacent in-person and telephonic assessments with Cohen’s kappa. Bland Altman analysis was used to assess differential bias in low and high score results.

**Results:**

Data from 74 patients were available for analysis. Floor effects were seen in the early post-injury time points (median: 3 (IQR: 0, 5) at 3 days post-enrollment) and ceiling effects in the late time points (median: 9 (IQR: 8, 10). Internal consistency was good to excellent with both in-person (Cronbach α: 0.91 (95%CI 0.88, 0.95)) and telephone administration (0.81 (0.73, 0.89). Temporal stability was also good (ICC: 0.83 (0.72, 0.89) in-person, 0.80 (0.68, 0.88) telephone). A strong linear correlation was found between in-person and telephone administration (Spearman’s ρ: 0.83 (CI: 0.78, 0.84), consistency was assessed as excellent (Cohen’s κ 0.81 (CI: 0.78, 0.84), and Bland Altman analysis showed no systematic bias.

**Conclusions:**

Telephone administration of the PSFS provides valid, reliable, and consistent data for the assessment of recovery from snakebite envenomation.

## Author summary

Snakebite envenomation is an important but neglected tropical disease that impacts millions of people worldwide each year. These bites lead to both death and permanent disability. As they occur in tropical and subtropical regions, they primarily impact people from low-income areas of the world. As potential new treatments are being developed, we must understand their potential benefit in humans before they can be widely disseminated. Performing these human studies requires the ability to determine how patients recovered with these treatments. Having people return for evaluation during recovery is difficult in these low-income regions. We evaluated the ability to use a telephone version of an already accepted measurement of recovery in snakebite, the Patient-Specific Functional Scale. This study demonstrates that using this telephone-administered measure is feasible, valid, and reliable. With the results of this study, we now have an important tool to easily measure recovery in areas where snakebite predominates. This tool will help snakebite envenomation researchers evaluate the potential benefit of new treatments and accelerate the process of bringing new effective treatments to those snakebite patients in the most need.

## Introduction

Snakebite envenomation is a neglected tropical disease that affects as many as 1.8 million people per year with the overwhelming majority of patients from low- and middle-income countries (LMICs). Although snakebite envenomation is responsible for an estimated 94,000 deaths annually, the burden of injury is also immense, as many of the survivors sustain permanent disability.[1–5] To date, almost no clinical trials have attempted to study the impact of treatment interventions on snakebite-caused disability.[6–10] However, researchers face substantial challenges to performing high quality trials, and research instruments used to assess disability and recovery must be both validated and practical to administer in low-resource settings.

An essential element of high-quality clinical research is the use of patient-centered outcome measures, such as patient reported outcomes (PROs). Currently, no practical, inexpensive, reliable, validated PROs exist that are appropriate for evaluating patients with snakebite envenomation.[11, 12] This impacts snakebite envenomation research, particularly in LMICs due to cost and logistical barriers to in-person administration of a PRO. The patient may need to take time off from work, pay for transportation, coordinate childcare, or navigate the innumerable barriers that already exist to access healthcare in order to participate in an in-person outcome assessment. The ability to use a valid, reliable outcome measure administered by telephone eliminates many of these challenges. With the widespread use of cellphones in LMICs, a telephone-administered, validated PRO would be an inexpensive and useful tool in future snakebite envenomation research. [13]

The Patient-Specific Functional Scale (PSFS) is a validated, patient-centered measurement tool that assesses a patient’s functional impairment regarding specific physical activities that the patient identifies as important. Patients report three to five activities or tasks that they are unable to perform or have difficulty with due to their illness.[14] The validity of the PSFS has been demonstrated in numerous studies, particularly in those related to musculoskeletal disease or injury.[15–19] The PSFS administered in person has also been validated in studies involving patients with snakebite envenomation to the extremities.[11, 20] In fact, the PSFS administered in-person is highly correlated with more complex assessment tools and is very responsive to changes in patient functional status over time.[20] The PSFS can logistically be performed by telephone, but no studies have validated telephone-administered PSFS compared to in-person administration.

A recent snakebite envenomation clinical trial used in-person PSFS as the primary outcome and recorded additional assessments with telephone administered PSFS.[21] This provides the opportunity to validate the telephone version against the in-person criterion standard. The purpose of our study is to determine if telephone administered PSFS has similar validity, reliability, and consistency to in-person administration in this snakebite envenomation population. We report the psychometric properties of telephone compared to the PSFS administered in-person, specifically looking at: (a) content validity, (b) internal structure and consistency, and (c) temporal stability and external validity.

## Methods

### Ethics statement

The current study is a secondary analysis. The parent study procedures were reviewed and approved by the Western Institutional Review Board (WIRB) and by the IRB responsible for each clinical site. The activities of the coordinating center were approved by the Colorado Multiple IRB (COMIRB). Written informed consent was obtained from all subjects prior to participation.

### Study design

We performed a secondary analysis of the “The efficacy of Fab antivenom versus placebo plus optional rescue therapy on recovery from copperhead snakebite envenomation: a randomized, double-blind, placebo-controlled clinical trial.” The complete methods of this trial and participant selection have been published previously, but are detailed in brief. [21]

### Patient selection

Patients age 12 years or older with mild or moderate copperhead envenomation were randomized to receive Fab antivenom (FabAV) or placebo. Patients with severe envenomation, envenomation proximal to the elbow or knee, more than one extremity involved, pregnancy, or presenting >24 hours after envenomation were excluded. All 72 patients from the modified intention-to-treat population of this study were included in the analysis.

### Instrument

The original scale used was the PSFS, which assesses a patient’s ability to perform an important physical activity or task and consists of three to five activities in a single dimension. As the performance of the three-activity and five-activity tools are similar, the three-activity PSFS was chosen for this trial. The answers are given on an 11-point scale (0 = Unable to perform activity to 10 = Able to perform activity at the same level as before the injury or problem). Higher values indicate improved function. At the initial assessment, patients were asked to list three specific activities of their choosing and rank their ability to perform them. They were asked the same questions at follow up assessments at particular time intervals to monitor if their physical function was still affected and to what degree.[14] The questionnaire served as a tool to quantify activity limitation and measure functionality.

### Data collection

In-person PSFS was performed on days 3, 7, 14, 21, and 28 post-envenomation (+/- 1 day). Telephone PSFS was performed on days 10, 17, 24, and >28 post envenomation (+/- 1). As the in-person and telephone PSFS were not obtained on the exact same day, we use the following nomenclature: T1 (days 7 and 10), T2 (days 14 and 17), and T3 (days 21 and 24). An independent Data Monitoring Committee oversaw study conduct, and on-site monitoring was performed by the sponsor’s clinical research associates.

### Analysis

We first compared the descriptive mean scores between in-person and telephone administration of the PSFS using a t-test comparison, including calculations for standard deviation. We then compared telephone to in-person administration of PSFS by evaluating the following scale psychometric properties: content validity, reliability, and external validity.

We assessed content validity by evaluating the proportion of patients with floor and ceiling effects over time. We assessed reliability by evaluating internal consistency, temporal stability, and association between instruments. Regarding internal consistency, we used Cronbach’s alpha to determine if in-person and telephone-administered PSFS produced similar results. We considered a Cronbach’s alpha value above 0.7 as good.[22] We then measured temporal stability to assess the instrument's variation in time and to verify the test–retest reliability of the instrument, with an Intraclass Correlation Coefficient (ICC) above 0.75 considered adequate.[23] To evaluate temporal stability using times when the patient would be expected to have achieved a stable state of recovery. [21] Thus, we used day 14 to day 21 for in-person administration (T2 to T3) and day 17 to day 24 for the telephone measurements (T2 to T3) to calculate the ICC.

We used a Bland-Altman analysis to assess agreement between in-person and telephone administered PSFS versions, plotting the difference between two paired measurements against the average of the two measurements. The resultant values were plotted, with a line indicating perfect agreement (i.e. zero difference), allowing a visual assessment of variation related to the size of the mean. Confidence intervals (i.e. limits of agreement) were also calculated using non-parametric methods. [24, 25] We assessed external validity of the telephone-administered PSFS scores in comparison to the in-person–administered PSFS, using Spearman correlation coefficients (ρ).

## Results

Our sample was composed of 72 adults from 13 centers. Fifty-two percent were male and 89% were over 18 years of age. The mean age was 43 (SD 17.6) years. A tendency toward floor effects could be seen in the total PSFS with a median score of 3 (interquartile range (IQR): 0–5; range 0–8). On the initial (day 3) assessment, 28% of the participants scored 0, indicating a severe activity limitation. Ceiling effects were more evident on follow-up days 24 and 28, as the median score of PSFS total was 9 (IQR: 8–10; range 5–10), and 48% of the participants scored 10, which indicated no self-reported limitation in the chosen activities.

### Internal consistency was good to excellent

**Fig 1:**
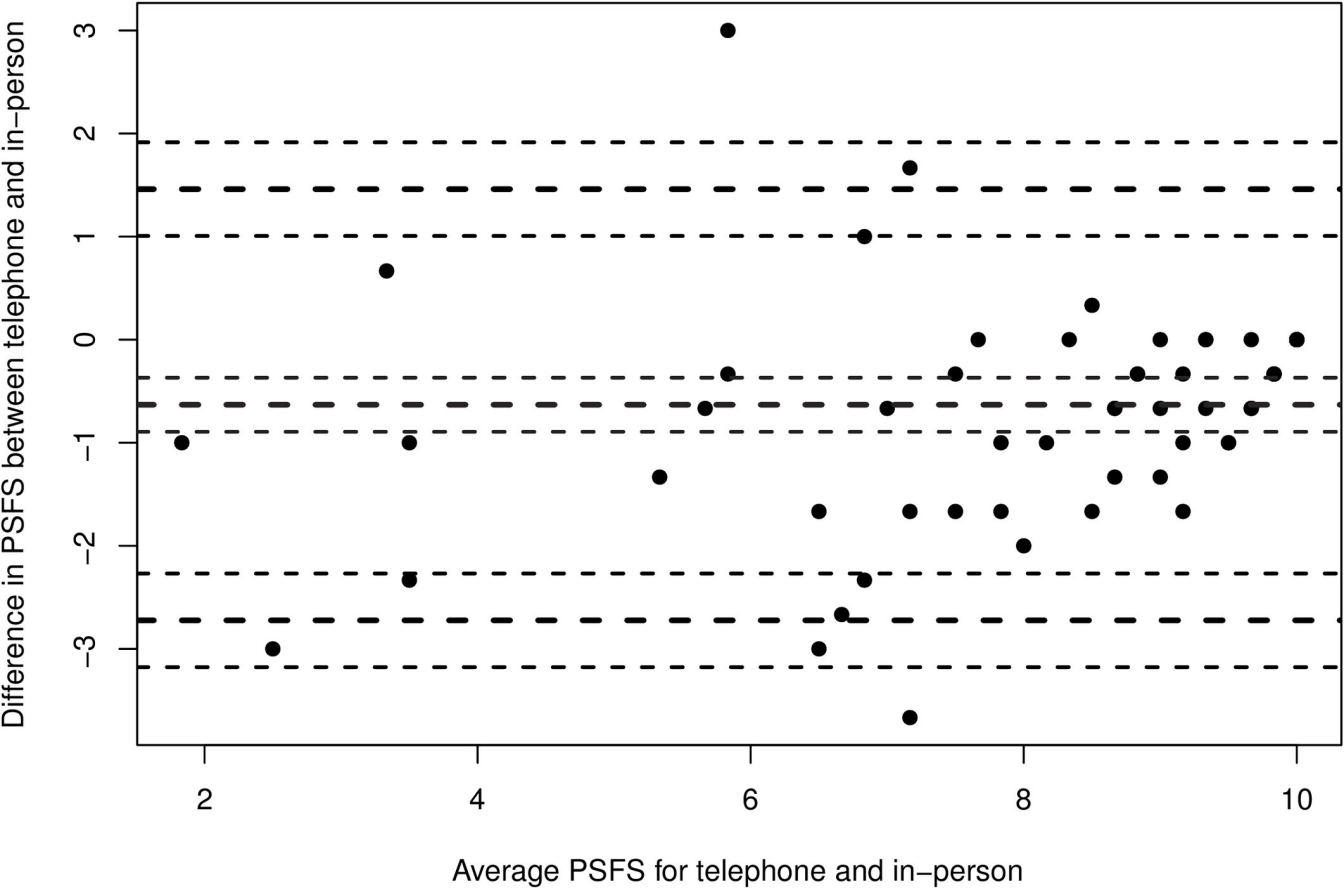
Bland Altman plot to display the difference between telephone and in-person administrations of the patient-specific functional scale.

Cronbach alpha values were above 0.9 (excellent) for in-person PSFS and between 0.7 to 0.9 (good) for telephone PSFS for all parameters (Table 1). The ICC confirmed good temporal stability of the scale, with ICC 0.83 (95% CI 0.72, 0.89) in-person and 0.80 (95% CI 0.68, 0.88) via telephone. Rating differences between in-person and telephone administered measurements ranged from -8 to 2, with a median difference of 0. Bland-Altman analysis found that 95% of the differences between paired ratings were between -4 and +1, with 50% of the differences between -2 and 0 (Fig 1). Overall there were only 4 sets of measures (5%) with differences of at least 6 points.

**Table 1:** Psychometric properties for content and construct validity.

	Snakebite Envenomation PSFS		
	In-person version	Telephone version	P-value
**PSFS, Mean (SD)**			
T1	5.37 (3.23)	6.62 (2.85)	0.01
T2	7.95 (2.22)	8.54 (1.92)	0.09
T3	9.12 (1.37)	9.40 (1.06)	0.18
**Floor effect, N (%)**			
T1	6 (8.4)	2 (2.8)	0.61
T2	0 (-)	0 (-)	-
T3	0 (-)	0 (-)	-
**Ceiling effect, N (%)**			
T1	9 (12.7)	11 (15.7)	0.71
T2	18 (25.4)	24 (36.4)	0.51
T3	35 (49.3)	39 (57.3)	0.63
**Reliability**			
Cronbach's α(95% CI)	0.91 (0.88;0.95)	0.81 (0.73;0.89)	
**Temporal stability**			
ICC (95% CI)	0.83 (0.72;0.89)	0.80 (0.68;0.88)	
**Reliability (in-person vs telephonic)**			
ICC (95% CI)	0.87 (0.79;0.92)		
Spearman’s ρ	0.83 (0.80;0.93)		

ICC = Intraclass correlation coefficient; CI = confidence interval

**Fig 2:**
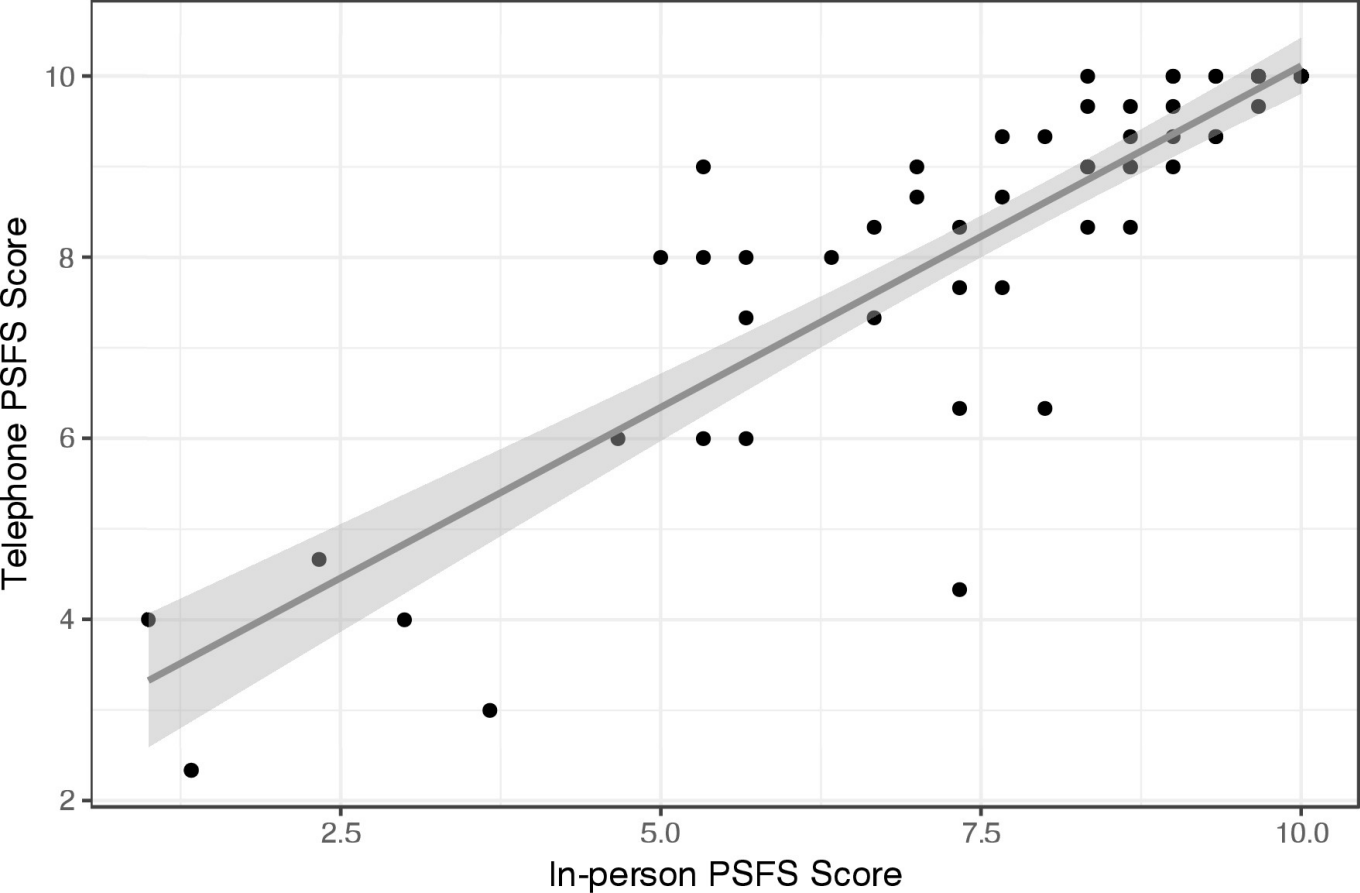
Spearman correlation coefficient demonstrating strong linear correlation between telephone and in-person administrations of the patient-specific functional scale.

The Spearman Correlation coefficient demonstrated a very strong linear correlation (>0.8) between PSFS administered in-person and telephone (ρ: 0.83 (95% CI 0.80, 0.93) (Fig 2). Comparison of the in-person and telephone administered PSFS over time shows a time dependent progression of PSFS regardless of method of administration (Table 1).

## Discussion

Based on multiple analyses, we determined that results obtained from in-person and telephone administrations of the PSFS are equivalent. Both scales exhibit a floor effect at earlier time points and a ceiling effect at later time points, signifying that patients who sustain copperhead snakebites have significant early functional disability and a good long-term recovery response. Both scales have acceptable (good to excellent) internal consistency and good temporal stability. ICC and correlation coefficient, which are a measure of between–scale reliability, also demonstrates very good association.

Telephone-based testing has been previously used as an alternative data collection method in clinical research. The use of telephone versus in-person PROs has been studied[26, 27], however, this is the first study looking into the PSFS as a telephone-administered PRO. These results are important as the PSFS has uniquely flexible characteristics that have not yet been evaluated with different administration methods. Our results suggest that the PSFS may function well in alternative methods of administration such as text messaging, online surveys, and mobile applications.

The internal consistency of the telephone administered PSFS in our study was excellent and comparable to the in-person version. Reliability of the telephone administered PSFS in snakebite envenomation is similar to the findings of prior studies performed using in-person PSFS in knee dysfunction, acute and chronic low back pain, cervical pain, chronic lateral epicondylitis, and chronic obstructive pulmonary disease (COPD).[14, 28–31]

The telephone administered PSFS demonstrated good stability over time, comparable to the in-person PSFS in snakebite envenomation. Additionally, its temporal stability is similar or better than the in-person PSFS in other injuries or diseases.[19] The existing literature has reported test–retest values ranging from 0.55–0.95.[16, 19, 30] These findings are concordant with the Bland Altman analysis which suggests that the telephone-administered PSFS is a reliable tool with minimal bias.

The external validity of the telephone administered PSFS against the in-person PSFS was also high and consistent with the literature on other PROs administered by telephone.[27, 32] However, the PSFS has the advantage of flexibility in patient choice of activities assessed. This adaptability of the instrument allows the outcome to be tailored to an individual patient and assesses function across a diverse population. In contrast to many other PROs that would be reasonable to use in snakebite research, the PSFS is not limb-specific. This allows for assessment of a wide range of patients, including those with upper extremity and lower extremity bites, with results that are broadly generalizable in snakebite envenomation.

Based on our results, we now have a PRO that is easily used in clinical research where in-person measurements are financially and logistically prohibitive. This is the case in global snakebite envenomation research, particularly in LMICs where this disease predominates.[5, 10] The PSFS is the most extensively validated PRO in snakebite envenomation research, and telephone administration makes this instrument pragmatic to use in real-world settings.[11, 12, 20] Given the potential use of the telephone administered PSFS, researchers should be aware that mobile phone penetrance is not universal, but its use has been recommended by the World Health Organization and the National Institutes of Health Fogarty Center [33–35]. Additionally, there are communities where information by phone could be biased due to the presence of authoritarian regimes, political violence or organized crime. Lastly, the telephone PSFS measures functionality which is one of the most important outcomes in snakebite envenomation. However, it does not measure other important patient-centered outcomes such as psychological sequela, disfigurement leading to stigmatizations, or others.

### Limitations

Our study has several limitations that could impact the interpretation of our results. First, this is a secondary analysis of an existing data set and not the primary hypothesis of the original study. Therefore, we could not directly compare the telephone to in-person PSFS administration at the identical time points as we were limited to the original data collected. Nevertheless, because our times points are very close to each other, the curves for PSFS administered in-person and by telephone overlap substantially and thus are very similar. Additionally, our study is specific to copperhead envenomation, which on average has less severe envenomation than other snakebites such as rattlesnakes or tropical venomous snakes.[36, 37] In generalizing our results to other snake species, there is risk that the early floor effects would be greater. However, these floor effects disappear over time during recovery. It is likely that these floor effects would also resolve with envenomation from other species during recovery, making the tool useful for assessing recovery at greater than 3 days.

### Conclusion

The telephone-administered PSFS is a valid and reliable PRO to assess functional recovery in snakebite envenomation compared to in-person administered PSFS.

## Supporting information

S1 Dataset Patient-specific functional scale scores reported in-person on days 3, 7, 14, 21, and 28 post-envenomation (+/- 1 day) and by telephone on days 10, 17, 24, and >28 post envenomation (+/- 1). (CSV)
